# Research progress of novel anti-tumor drug formulations

**DOI:** 10.3389/fonc.2024.1507958

**Published:** 2024-12-16

**Authors:** Yan Liu, Qie Guo, YunYan Shi, MengNa Cui, FanBo Jing

**Affiliations:** ^1^ Department of Pharmacology, School of Basic Medicine, Qingdao University, Qingdao, China; ^2^ Department of Clinical Pharmacy, The Affiliated Hospital of Qingdao University, Qingdao, Shandong, China

**Keywords:** cancer, anti-tumor drug, nano-drug delivery systems, new formulations, nanomedicine

## Abstract

Cancers have become the second leading cause of death worldwide, following cardiovascular diseases.Traditional anti- cancer strategies, including radiotherapy chemotherapy, surgery, and targeted therapies, have been widely used but are often reassessed due to their significant side effects and relatively low cure rate. Recently, the development of novel formulations for anti-tumor drugs has gained considerable attention, marking a pivotal step forward in cancer treatment advancements. These innovative formulations aim to enhance the therapeutic efficacy of anti-tumor drugs by employing advanced drug formulation technologies and delivery systems. In particular, nano-drug delivery systems (NDDS) have emerged as a promising approach to improve drug targeting, reduce side effects, and overcome drug resistance. This review highlights recent progress in NDDS for anti-tumor drug development and explores the future prospects of these advanced formulations in improving cancer treatment outcomes.

## 1 Introduction

Cancer remains one of the leading cause of death worldwide, accounting for over10 million mortalities annually. Various factors contribute to its onset, including aging, alcohol consumption, obesity, smoking, radiation exposure, carcinogens, chronic inflammation, genetic mutations and immune suppression (1). Significant progress in anti-tumor treatments, particularly in drug development, immunotherapy, gene therapy and RNA Interference (RNAi) therapy, has led to a deeper understanding and more effective approaches to addressing the challenges posed by cancer (2). Key advancements include chemotherapy drugs and molecular targeted drugs, immune checkpoint inhibitors (ICIs) in immunotherapy, the CRISPR/Cas system in gene therapy, and microRNAs (miRNAs)-based RNAi therapies. These innovations have significantly improved patient survival rate, particularly when cancers are detected early and treated effectively (3).

Nanomedicine refers to drugs prepared through nanotechnology, and one way to improve the solubility of insoluble drugs is to make them at nanoscale (4). The advent of nanocarriers has infused new vitality into cancer therapy,driving continuous advancements in the field. Nanocarriers such as polymer micelles, liposomes, nanoemulsions, dendrimer macromolecules and inorganic nanoparticles have been extensively utilized to address limitations in traditional cancer therapies (5). These nanocarriers provide unique advantages,including controlled and flexible drug release, enhanced tumor immune response, increased sensitivity to drug therapies, reduced systemic side effects, and improved therapeutic efficacy (6).

In recent years, innovative anti-tumor agents, particularly nanocarrier-based systems have expanded the arsenal of cancer treatments. They enhance the effectiveness of anti-tumor drugs, immunotherapies, gene therapies, and RNA interference therapies while minimizing adverse reactions. This review highlights recent advancements in the application of nanocarriers in cancer therapy. By exploring their roles in enhancing treatment outcomes, we aim to inspire further research into the potential of nanocarriers to revolutionize cancer treatment.

## 2 New anti-tumor drug dosage forms: the backbone of chemotherapy drugs

**Figure 1:**
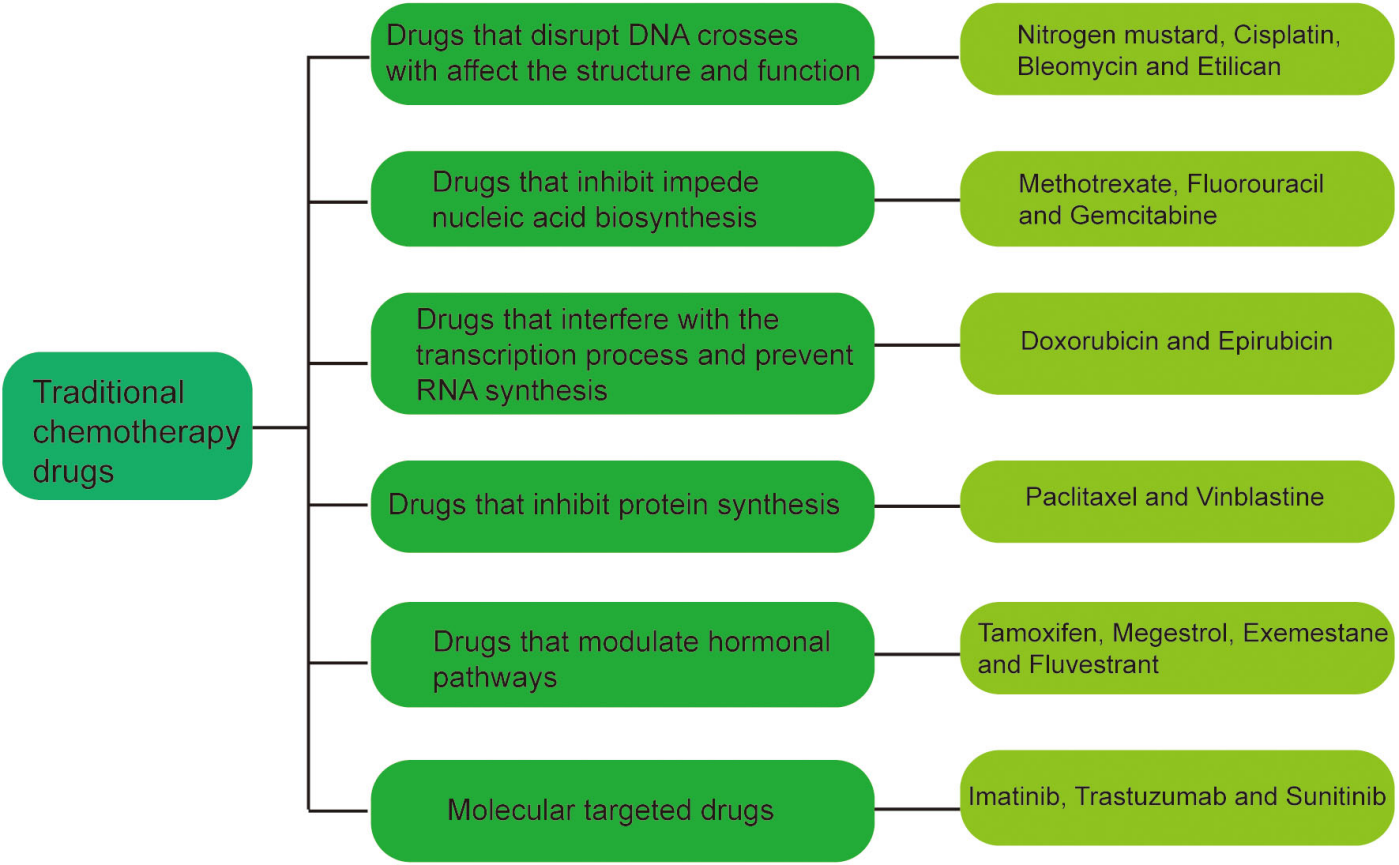
Classification of traditional chemotherapeutic agents.

At present, the known traditional chemotherapy drugs can be roughly divided into the following seven categories (**Figure 1**) (1): Drugs that disrupt DNA structure and function, such as nitrogen mustard, cisplatin, bleomycin and Etilican (2); Drugs that inhibit nucleic acid biosynthesis, such as methotrexate, fluorouracil and gemcitabine (3); Drugs that interfere with the transcription RNA synthesis, such as doxorubicin and epirubicin (4); Drugs that inhibit protein synthesis such as paclitaxel and vinblastine (5). Drugs that modulate hormonal pathways such as tamoxifen, megestrol, exemestane and fluvestrant (6); Molecular targeted drugs, such as imatinib, trastuzumab and Sunitinib. These drugs are administered through various routes,including oral administration, injection, intravenous infusion, subcutaneous implantation, and localized delivery, depending on the specific drug and treatment protocol.

Although these drugs have a killing effect on cancer cells, they can also produce a range of adverse effects and harms on normal cells. Firstly, traditional chemotherapy drugs can kill rapidly multiplying cells, including cancer cells. However, they also affect cells with toxic effects that normally divide rapidly, such as blood-forming cells, intestinal mucosal cells, and hair follicle cells, causing discomfort such as anemia, iron (7) deficiency, nausea, vomiting, diarrhea, and hair loss. Secondly, chemotherapy suppresses immune function, increasing vulnerability to infections. Patients may develop severe conditions like cystitis, necessitating antibiotic treatments (8). Additionally, other side effects include extreme fatigue, caused by the toxicity of the medication as well as the psychological stress of treatment (9). Nausea and vomiting particularly common, often leading to malnutrition due to decreased appetite and weight loss (10). Otherwise, ototoxicity, nephrotoxicity, and hepatotoxicity seems not to be exceptional. Some medications may cause damage to hearing, resulting in hearing loss. They can also have toxic effects on the kidneys and liver, leading to kidney function or liver dysfunction (11). Certain chemotherapy drugs also cause neurological problems such as peripheral neuropathy, paresthesia, and weakness of limbs. They also cause cardiotoxicity, fertility and sexual toxicity. Most importantly, these long-term side effects can last for many years, with lasting effects on a patient’s quality of life.

An additional challenge is drug resistance, a significant obstacle in chemotherapy.Drug resistance refers to the gradual decrease in the sensitivity of tumors to chemotherapy drugs, which is an important reason for the failure of tumor chemotherapy, and also an urgent problem to be solved by cancer chemotherapy (12). Drug resistance to chemotherapeutic drugs can be divided into intrinsic resistance and acquired resistance. The epigenetic alterations contributed to the development of intrinsic drug resistance. However, acquired drug resistance develops gradually in the process of drug therapy, that is, the drug resistance obtained by tumor cells after longterm small doses of cytotoxic drugs. The underling mechanisms promoting acquired drug resistance were shown as follows (1):Reduced accumulation of chemotherapeutic drugs in tumor cells (2); Decreased cellular uptake of drugs (3); Lowered levels or activity of drug-activating enzymes (4); Increased levels or activity of drug-inactivated enzymes (5);Increased content of drug rake (6); Reduced availability of substrates necessary for drug action (7); Activation of alternative metabolic pathways, such as anti-metabolic drug resistance (8); Enhanced DNA repair capacity in tumor cells, such as alkylating agent resistance.

Alternatively, chemotherapy drugs also have many disadvantages, including high toxicity, significant cost, variability in individualized treatment responses, pain from injections, and risks of infection (13), and the side effects caused by these reasons are very harmful to human body. We are eager to develop new anti-tumor drug formulations that will minimize the side effects of chemotherapy drugs without compromising their efficacy. In order to overcome these challenges, the research and development of anti-tumor drugs has made great progress, a variety of new drugs, new dosage forms continue to emerge, they provide us with the theoretical basis for research, but also help us to better understand the development of anti-tumor drugs and the status quo, bring hope for cancer patients.

**Figure 2:**
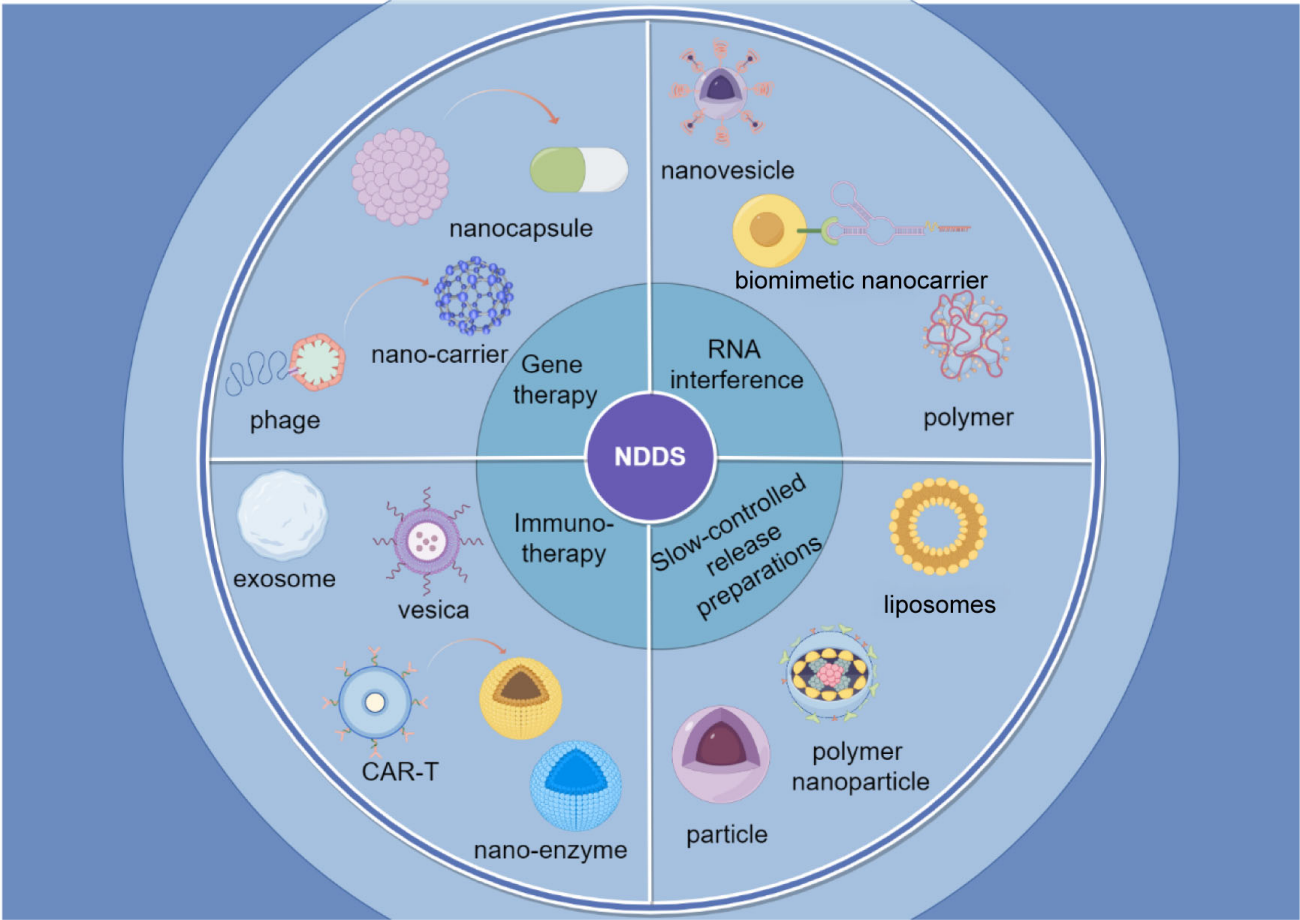
Classification of nano-drug delivery systems. The figure shows a brief introduction to four applications of nanomedicine delivery systems and their forms of application.

Common nanocarriers, such as the self-assembled nanomedicine combined with berberine derivatives and doxorubicin, can enhance anti-tumor and anti-metastasis efficacy through mitochondrial pathway. It has been found that these nanocarriers loading chemotherapy drugs can significantly inhibit tumor cell proliferation and lung metastasis (14). Similarly, D-alpha-tocopherol polyethylene glycol 1000 succinate (TPGS) modified carboxymethyl chitosan rhodopsinin (TCR) polymer micelles (pmms) for oral administration of paclitaxel, and demonstrated that the micelles can significantly improve the oral bioavailability of paclitaxel (15). **Figure 2** provides a simplified classification of these nanocarriers. **Table 1** is a list of FDA-approved nanomedicines.

**Table 1:** FDA-approved nanoformulations.

Nanomedicine	Type	Therapeutic Use	Comments
Doxil	Liposome	Cancer (ovarian, multiple myeloma, etc.)	First approved liposomal drug; reduces cardiotoxicity
Abraxane	Nanoparticle-bound albumin	Breast, pancreatic, NSCLC	Enhanced solubility of paclitaxel, higher tumor uptake
Onivyde	Liposome	Pancreatic cancer	Improved drug stability and tumor targeting
Vyxeos	Liposome	Acute myeloid leukemia (AML)	Improved curative effect and survival rate
Marqibo	Liposome	Acute lymphoblastic leukemia	Enhanced targeting and reduced side effects
DepoCyt	Liposome	Neoplastic meningitis	Improved targeting and reduced systemic toxicity

In general, nanocarriers enhance chemotherapy efficacy, and improve drug stability. However, challenges such as high costs, safety concerns, and potential toxicity remain unresolved, necessitating further research to fully realize their potential. 

## 3 Nanocarriers for immunotherapy: a rising star in chemotherapy drugs

**Figure 3:**
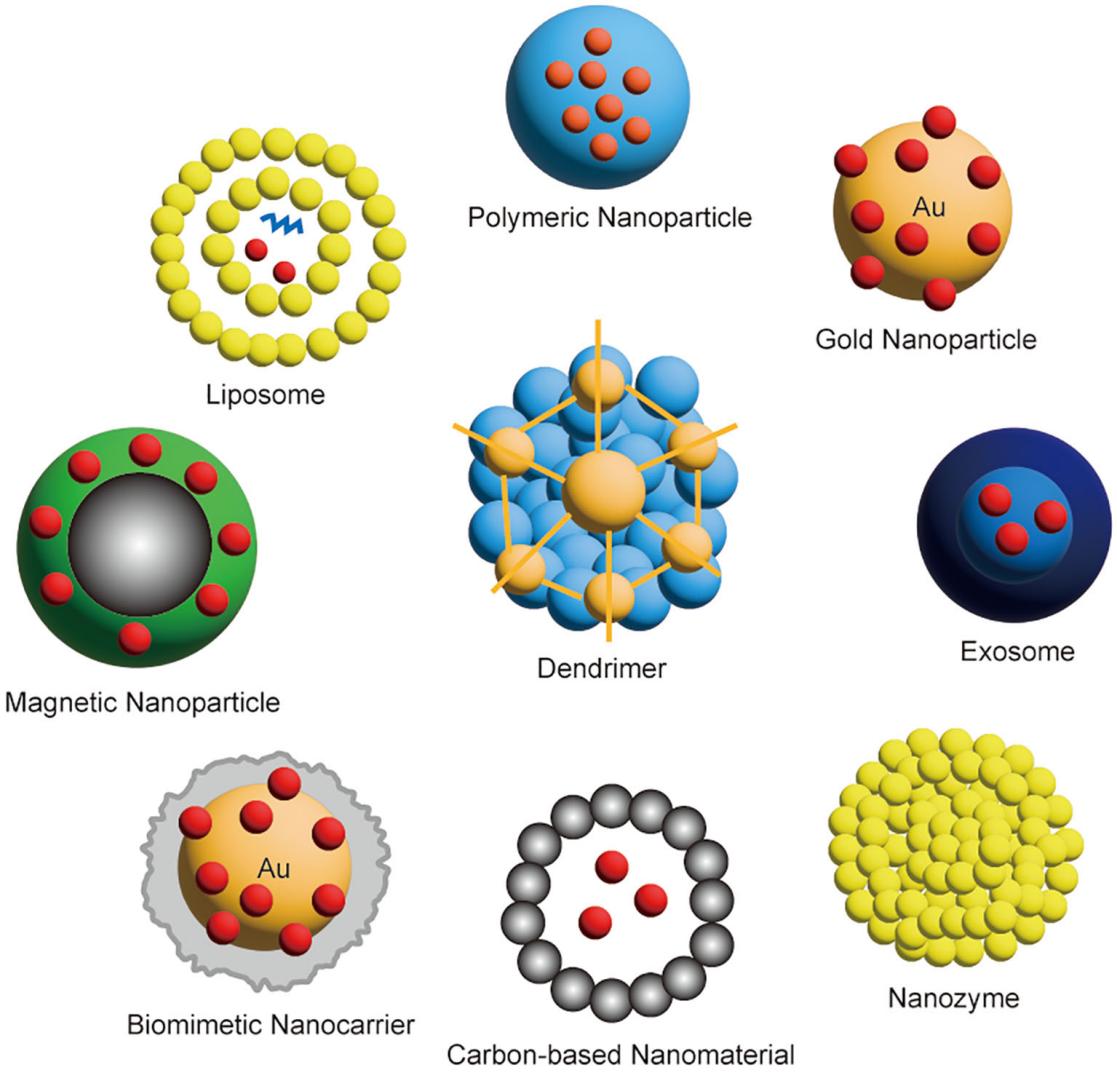
Classification of nanocarriers for immunotherapy.

Among cancer immunotherapies, the most common are interleukin, tumor vaccines and ICls such as death protein-1 (PD-1) and cytotoxic T-lymphocyte-associated protein 4 (CTLA-4) inhibitors as well as Chimeric Antigen Receptor T-Cell Immunotherapy (CAR-T) cell therapy. ICls are able to unblock the immune system from cancer cells, allowing the immune system to recognize and attack cancer cells more effectively. CAR T cell therapy, on the other hand, involves collecting T cells from a patient and then modifying these cells to carry a protein called chimeric antigen receptor (CAR), which allows them to recognize and kill tumor cells more effectively. It has the advantages of good therapeutic effect, low adverse reactions and low recurrence rate (16). The carrier of the immunotherapy nanoagent is shown in **Figure 3**.

For example, tumor-derived microparticles (T-MPs) can induce macrophages to release C-C motif ligand 2 (CCL2), a key chemokine. T-MPs can be used as tumor vaccine. It is mainly taken up by monocytes and macrophages, and induces CCL2 up-regulation of macrophages to stimulate strong tumor immune response (17);CAR-T cell therapy is a new breakthrough in cancer therapy. CAR can induce T cells to recognize and eliminate specific target antigens on the surface of cells (18). The binding of CAR to the target antigen expressed on the surface of cells does not depend on the membrane structure protein (MHC) receptor embedded in the lipid bilayer. Thus leading to strong T cell activation and powerful anti-tumor response (19), the method is suitable for refractory or recurrent B-cell precursors acute lymphoblastic leukemia, breast cancer, pancreatic cancer, thyroid cancer, brain cancer, etc. (20); Anti-targeting programmed cell death protein-1 or its ligand (anti-PD-1/PD-L1) inhibitor blocking combination strategy, studies have found that gold by blocking PD-1/PD-L1 is not enough to stimulate anti-tumor immune response, need to combine radiotherapy and chemotherapy, targeted therapy and other cancer treatment methods to achieve better anti-tumor effect, can reduce drug resistance (21); Exosomes are small vesicles with a diameter of about 30 to 150 nm that are secreted by cells and contain proteins, nucleic acids, and other bioactive molecules encapsulated by a lipid bilayer of the cell membrane (22). The principle of treating tumors is to use it as a natural cell-to-cell communication carrier to play a role in regulating the tumor microenvironment (23), regulating immune responses, and transmitting treatment information, so as to achieve the treatment and regulation of tumors (24); Nanozyme is an enzyme nanomaterial prepared by nanotechnology, which has high stability, biocompatibility and specificity. The principle of treating tumors is to use their special biocatalytic properties and targeting properties to regulate specific biological responses or physiological processes in the tumor microenvironment, so as to achieve the treatment and regulation of tumor cells (25); Cytokine gene vaccine is a tumor immunotherapy method that uses genetic engineering technology to introduce genes encoding specific cytokines into the patient’s body to activate the patient’s own immune system to attack the tumor (26). Commonly used cytokines include tumor necrosis factor (TNF), interleukin (IL), interferon (IFN) (27), etc. These cytokines are able to regulate the proliferation, differentiation, activation, and function of immune cells, thereby enhancing the immune system’s ability to recognize and tumor ablation (28).

Nanomedicine has demonstrated promising potential in enhancing immune response rates and reducing immune-related adverse events (irAEs). For example, Jinsheng Huang et al. combined sonodynamic therapy (SDT) with anti-PD-L1 antibody (aPD-L1) immunotherapy. This combination promoted tumor invasion inhibition, activated cytotoxic T cells (CTLs), and significantly reduced irAEs, effectively suppressing melanoma growth and postoperative recurrence (29). Immune checkpoint blockade (ICB) antibodies, such as aPD-L1, are effective in activating CTLs to combat cancers but show limited efficacy in prostate cancer (PCa). To address this, Yiyao Wang et al. developed a tumor acidity-activatable macromolecular nanodrug (P-PDL1-CP) featuring a PDPA core and polyethylene glycol (PEG) coatings conjugated with aPD-L1. This nanodrug achieved notable therapeutic effects in PCa with minimal side effects (30). Additionally, Bin Xu et al. designed an acid-activated poly(amino acid)-calcium phosphate hybrid nanomotor (PCaPmotor) for CaP mineralization, incorporating mPEG-Pasp-PPhe@THZ531 micelles (Poly@THZ) and αPD-L1 antibodies. This PCaP@THZ/αPD-L1 system enhanced immunotherapy efficacy while minimizing side effects (31).

As an burgeoning technology in cancer treatment, nanocarrier-mediated immunotherapy holds tremendous potential for development. However, further research is required to address individual variability and optimize therapeutic strategies.

## 4 Gene therapy via nanocarriers: an strategy cannot be left behind in cancer therapy

**Figure 4:**
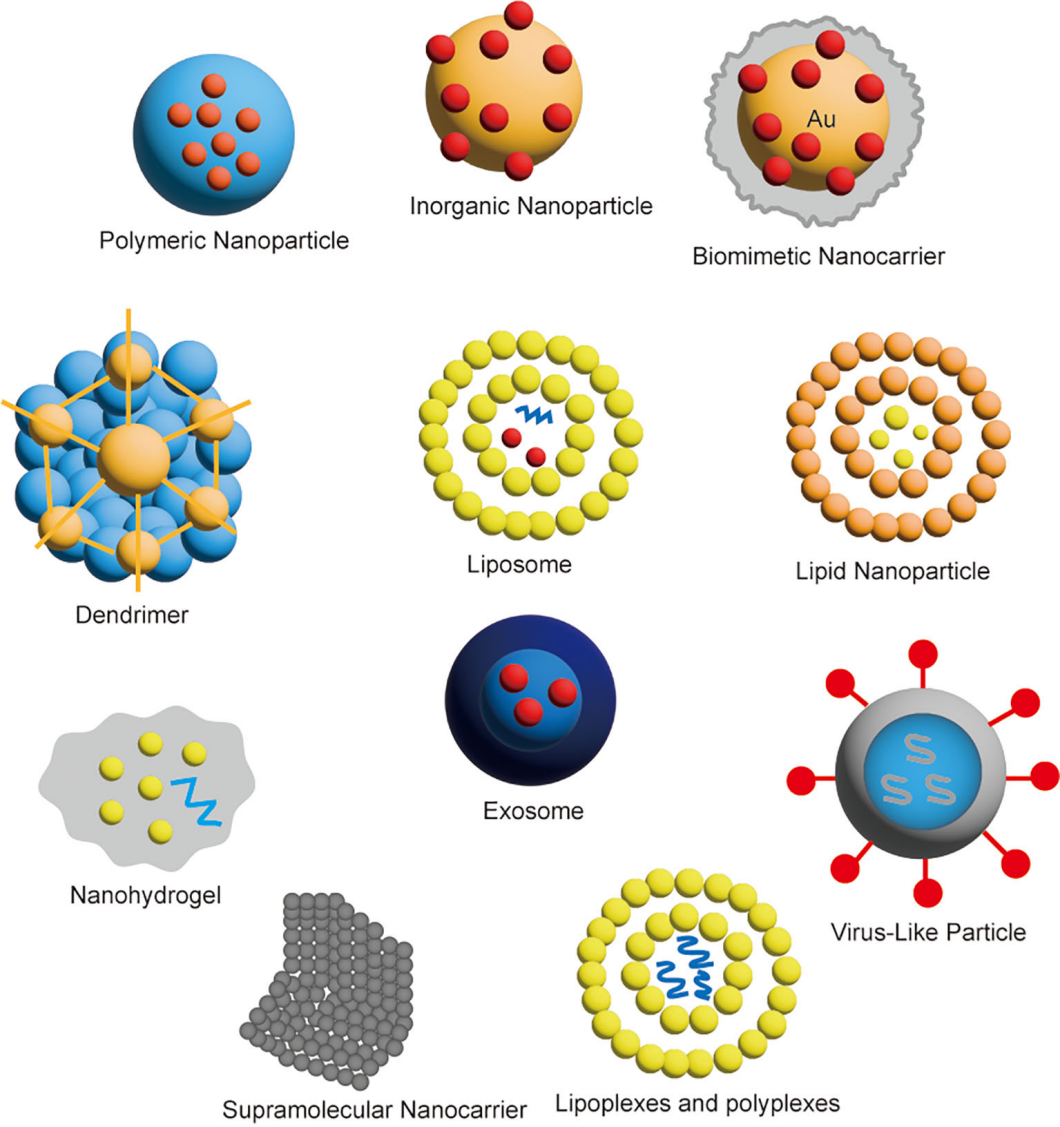
Classification of nanocarriers for gene therapy.

Gene therapy is an experimental personalized approach cancer treatment that modifies a patient’s genes or introduces carriers to repair or suppress tumor-related genes. The basic principle is to treat disease by introducing, modifying, or regulating genes in the patient’s body to correct certain genetic defects, alter the expression of abnormal genes, or enhance the function of normal genes. Gene therapy offers the potential for targeted tumor destruction with greater selectivity and safety compared to traditional chemotherapy (32). The nanocarriers used for gene therapy are shown in **Figure 4**.

For example, phage-mediated cancer gene therapy has improved the safety and effectiveness of tumor therapy by using phage carriers as systematic delivery carriers of therapeutic genes and drugs (33); miRNA gene editing for cancer is an emerging therapeutic method, which is based on the regulation of miRNA expression levels in tumor cells, thereby affecting tumor growth, metastasis, and drug resistance. The CRISPR/Cas gene editing system, where immune-related genes have emerged as promising targets for therapeutic genome editing (34). CRISPR-Cas is a prokaryotic immune system that endows prokaryotes with resistance to foreign genetic material (35); And neoantigen t cell receptor gene therapy for pancreatic cancer, in which a patient with progressive metastatic pancreatic cancer was genetically engineered with T cells to clone two HLAC*08:02 restrictive T cell receptors (TCRs) that target the tumor-expressed mutant KRAS G12D. It was found that the patient’s metastases subsided, the response was sustained at 6 months, and the tumor objective regression after 6 months (36); Cas9 belongs to the type ii CRISPR system and is the currently most widely used gene-editing tool (37). Some researchers have designed a special nanocapsules, single CRISPR-Cas9 nanocapsules for glioblastoma gene therapy, which can cross the blood-brain barrier and improve the efficacy (38).

Jinsheng Huang et al. developed a vitamin A (VA)-modified crosslinking nanopolyplex, termed T-C-siRNA. This system features a negative charge, retinol-binding protein (RBP) hijacking, and cytoplasmic microenvironment-responsive siRNA release. By leveraging dual sensitivity to reactive oxygen species (ROS) and cis-diol compounds, this approach enables targeted cytoplasmic siRNA delivery. The platform holds promise for RNAi therapies targeting inflammatory diseases and malignancies (39). Xinghua Huang et al. introduced novel cation-free siRNA-cored polymeric nanocapsules, which demonstrated efficient siRNA encapsulation, high stability, and *in vivo* tumor-targeted gene silencing without cation-related side effects (40). Additionally, they synthesized an mPEG-P(Asp-co-TkCPT) copolymer linked to camptothecin (CPT) via a ROS-sensitive dithioketal (Tk) linker. These self-assembled PTkCPT micelles served as nanotemplates for calcium phosphate (CaP) mineralization. By blocking ADP-ribosylation factor 6 (Arf6) signaling, the nanoplatform induced mitochondrial aggregation and an ROS burst, leading to oxidative catastrophe and enhanced chemotherapy through CPT release. This strategy significantly inhibited tumor growth (41).Gene therapy, as an innovative cancer treatment, offers high precision and targeted capabilities that minimize tissue damage. However, challenges related to tolerability and safety remain, underscoring the need for further research to optimize these therapeutic platforms.

## 5 RNA interference and nanocarriers: a note of amusement in cancer therapy

**Figure 5:**
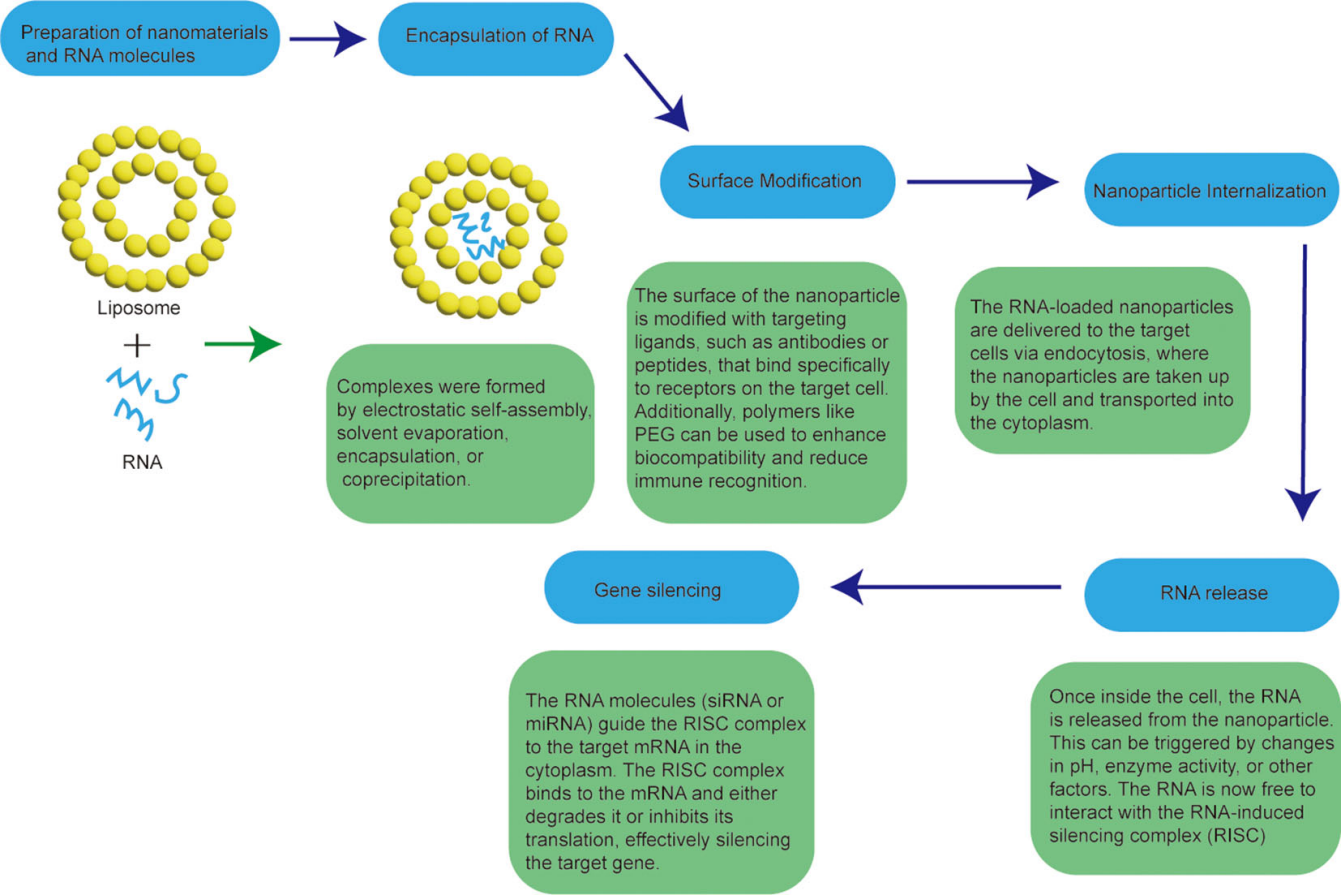
Workflow for the preparation of nanoformulations for RNA interference.

RNAi is a molecular biology technique that utilizes RNA molecules to regulate and silence gene expression. The technique was first discovered by studying gene silencing in plants, and later found to exist in mammalian cells (42). The basic interfering technique involves interfering with specific RNA sequences of the target gene using small interference RNA or miRNA molecules (43). These small Rnas are able to partially complement and combine with the target RNA sequence to form an RNA-induced silencing complex (RISC, RNA-induced silencing complex), which results in the degradation or translation obstruction of the target RNA, thereby inhibiting the expression of the gene (44). The advent of RNA-mediated gene suppression technology is a turning point in the field of RNA biology. The key regulatory roles of different Rnas in multiple cancer pathways make them rich targets and innovative tools for developing anti-cancer therapies (45). Identification of antisense sequences, short interfering Rnas (SiRnas), miRNAs, anti-miRs, and mrNa-based platforms holds great promise in preclinical and early clinical evaluation of LCS (46). The preparation process of nanoformulations for RNA interference is shown in **Figure 5**.

For example, Eukaryotic Translation initiation Factor 4E (eIF4E) is a key translation initiation factor involved in directing ribosomes to the 7-methylguanosine 5 ‘cap structure of mRNA and plays a critical role in some cellular processes. Studies have shown that overexpression of eIF4E can induce tumorigenic transformation in various cell lines, and it is dysregulated in approximately 30% of human cancers, including colorectal, prostate, lung and breast cancers. Currently eIF4E has been identified as a target for the treatment of colorectal cancer (47); Exosomes are naturally occurring nanovesicles that display various adhesion proteins on their surface, enabling them to interact with cell membranes and offering high biocompatibility. Some researchers have used this property in gene therapy for siRNA. It is used to silence the RAD1 gene, which is one of the main targets for cancer treatment (48). Liposomes are involved in drug delivery as carriers of RNA interference technology, and some researchers use vascular endothelial growth factor receptor 2 (VEGFR2) monoclonal antibody (mAb) modified photoresponsive liposomes to encapsulate DNA, because the liposomes are photosensitive and will degrade under light. The presence of VEGFR2 monoclonal antibodies allows specific targeting of the VEGFR2 receptor (49). This specific, controlled release maximizes tumor targeting ability and improves therapeutic efficiency.

RNAi holds great promise as a cancer treatment strategy. Some progress has been made in developing clinically viable RNAi delivery systems, particularly liposome, peptide-based, and polymer systems (50). However, challenges related to stability and delivery still need to be addressed in further research.

## 6 Slow and controlled release formulations of nanocarriers

Slow-controlled release formulations of anti-tumor drugs play a significance role in cancer treatment. These formulations offer sustained drug release, improving therapeutic efficacy, reducing drug dosage, minimizing toxicity, and providing more convenient treatment regimens. Additionally, slow-controlled release formulations are not significantly affected by factors such as pH, enzymes, and gastrointestinal motility. Sustained release refers to the gradual release of the drug into the body, maintaining an effective blood concentration over an extended period (51). This release process typically follows first-order or Higuchi diffusion kinetics. Intravenous injections, commonly used in systemic chemotherapy, can lead to adverse effects on healthy cells, such as pain at the injection site, repeated injections, potential infections, and the need for hospitalization, all of which can negatively affect the patient’s mood (52). The nano preparation has intelligent and prolonged drug release performance, and can be made into a slow-release preparation to reduce the number of injections and the toxic effect on healthy cells, thus improving the drug efficacy and improving the patient’s mood (53); Controlled release formulations refer to formulations in which drugs are slowly released at or near constant speed according to pre-set procedures, generally in line with zero-order kinetic processes. It is designed to provide a more efficient and controlled drug delivery system. These formulations can achieve controlled release of drugs in different ways.

**Figure 6:**
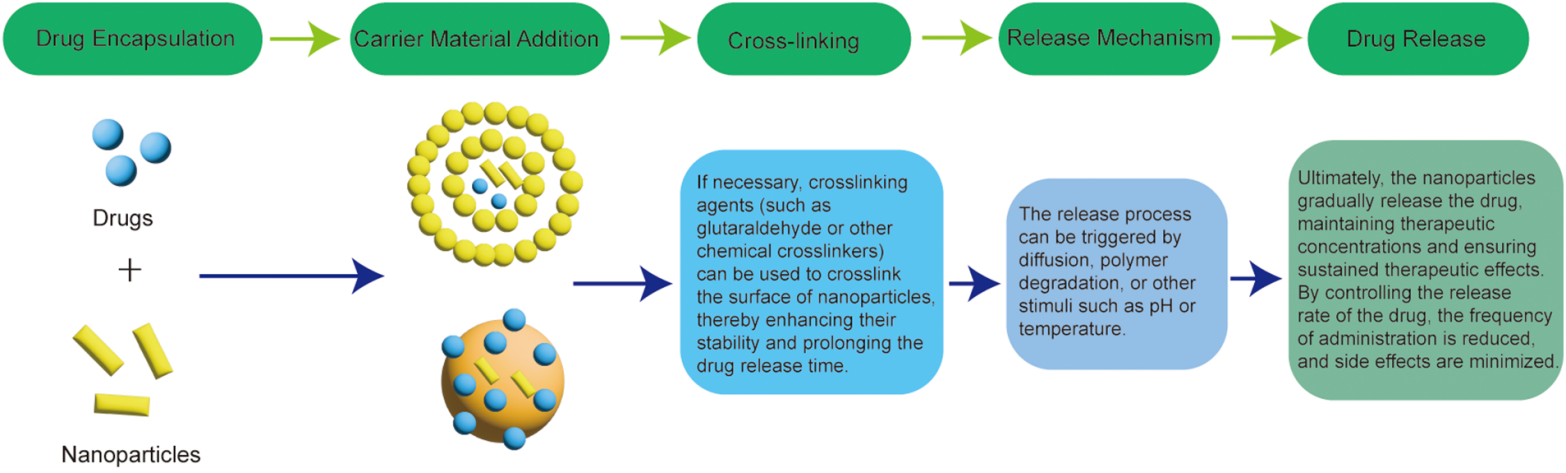
Process for the preparation of nanoformulations for sustained-release formulations.

Slow controlled release formulations have the following advantages (1): Reduced frequency of administration: For drugs with a short half-life or those requiring frequent dosing, these formulations reduce the number of administrations, improving patient compliance (2). Stable drug concentration: These formulations maintain stable blood drug levels, avoiding peaks and troughs, which helps minimize toxic side effects and reduce the likelihood of drug resistance (3). Optimized therapeutic effects: Slow-controlled release formulations ensure that drugs achieve their maximum therapeutic potential (4). Customized release: The release of drugs can be timed and targeted based on treatment requirements, making these formulations well-suited for disease-specific therapies (2). The dosing schedule cannot be adjusted flexibly (3). The equipment and process costs are expensive. The formulation process of the nanoformulation for the sustained-release formulation is shown in **Figure 6**.

Common types of slow-controlled release formulations include particles, liposomes, polymer nanoparticles, etc. For example, chitosan-triphosphate nanoparticles, ph-sensitive nanocomposites are synthesized through the electrostatic interaction between positively charged chitosan nanoparticles and negatively charged graphene (BSG), which is commonly used for the inclusion of anti-cancer drug azithromycin (DOX). Finally, the experimental results showed that the DOX released from the nano-composite had a significant inhibitory effect on tumor division (54); Doxorubicin hydrochloride liposome injection is the first clinically approved liposome preparation for the treatment of solid tumors, transplantable leukemia and lymphoma (55); Some researchers used PEG conjugated Fe3O4 nanoparticles as a sustained-release doxorubicin anticancer vector, which has been proven to have anticancer potential, cancer targeting ability and ROS generation ability, and has effective drug coating and sustained-release ability in chemotherapy (56).

In general, anti-tumor drug sustained-release formulations have many benefits in improving the therapeutic effect, reducing side effects and providing convenient treatment options. These formulations are of great significance in improving the success rate of tumor treatment and the survival rate of patients, but at the same time, more research and clinical practice are needed to optimize their application.

## 7 Biomimetic medicines and related clinical status

**Figure 7:**
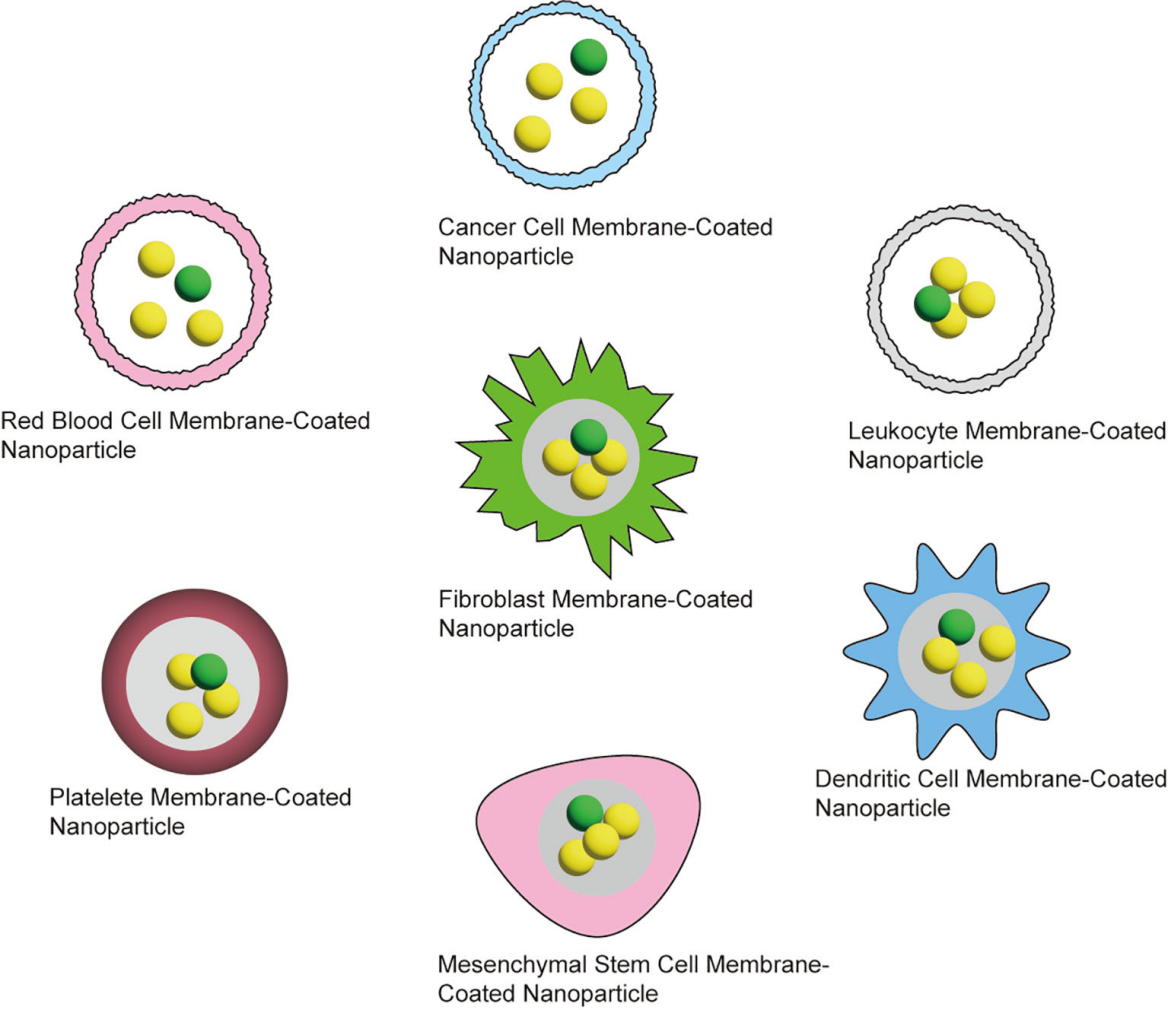
Various carriers for biomimetic nanomedicine.

Nanotechnology has significantly advanced the diagnosis and treatment of tumors. Despite the numerous technical advantages of nanomedicine, the clinical translation of nanoparticles (NPs) has been hindered by several delivery limitations, such as poor targeting and rapid clearance from the body (57). To overcome these challenges, biomimetic approaches involving immune cell membranes have been proposed as a solution. Biomimetic nanomedicine involves nanomaterials that replicate the structure and function of biological systems or biomolecules, specifically designed for targeted therapy, drug delivery, and other medical applications. These materials enhance therapeutic efficacy by mimicking the natural structures and functions found within the body (58). Biomimetic carriers include various types of cell membranes, such as leukocyte/platelet hybrid membranes, erythrocyte membranes, cancer cell membranes, mesenchymal stem cells, macrophage membranes, and even 4T1 membrane-coated nanozymes. These carriers utilize the natural properties of immune and other cell membranes to improve targeting, drug delivery, and therapeutic outcomes (59). There are various carriers regarding the biomimetic nanomedicine as shown in **Figure 7**.

Rajendra Prasad’s team introduced cancer cell-derived membrane nanobubbles (CCMVs) integrated with gold nanorods (AuVNRs) as a highly specialized nanotherapeutic platform. These CCMVs exhibit deep tumor diffusion, excellent biocompatibility, and enhanced tumor targeting capabilities (60). In a study by Deepak S. Chauhan et al., gold-deposited plasmonic polylactic-co-glycolic acid nanoshells (AuPLGA NS)-modified graphene oxide (GO) nanosheets (GO-AUPLGA) and NIR dye (IR780)-loaded GO nanosheets (GO-IR780) were successfully prepared *in vitro*. The study showed that independent, complete photothermal ablation of cancer cells occurred after just 4 minutes of exposure to an 808 nm NIR laser, without the need for any chemotherapeutic agents. Notably, the GO-AuPLGA system demonstrated significant potential for delivering repetitive photothermal therapy to large tumors while minimizing damage to surrounding healthy cells (61). Nanoengineered photoactive therapeutic diagnostic agents are an emerging technology for the treatment and diagnosis of cancer. Examples include photodynamic therapy (PDT) and photothermal therapy (PTT) (62), wherein photoactivating agents release active substances or generate heat within tumor cells, thereby destroying cancer cells. Moreover, these nanotherapeutic agents can be utilized for imaging purposes, facilitating early diagnosis and treatment monitoring. This technique enhances the therapeutic effect by specifically targeting cancer cells while minimizing impact on healthy tissues (63).Biomimetic nanomedicines offer significant theoretical advantages, particularly in targeted therapy, reducing side effects, and improving drug efficacy. However, challenges such as high manufacturing complexity, production hurdles, and potential immune responses currently restrict their clinical application, limiting them to the research and development stage. In contrast, traditional nanomedicines, though less advanced in these aspects, continue to be widely used in clinical practice due to their established production methods and lower costs. With ongoing advances in technology and novel materials, biomimetic nanomedicines are expected to play a larger role in future cancer therapies.

## 8 Challenges and future prospects

Cancer is currently the leading cause of morbidity and mortality worldwide.Although chemotherapy and other treatments can i slow tumor growth, traditional chemotherapy methods have significant limitations, often resulting in clinical failure. The development of novel anti-tumor agents faces numerous challenges but also holds promising prospects for the future.

One of the most urgent goals is to enhance the precision of drug delivery while minimizing damage to healthy cells. Additionally, improving the stability of of these drugs is crucial, ensuring that they remain at effective concentrations for extended periods. More importantly, overcoming immune suppression and drug resistance is essential for the success of nanocarriers-based cancer therapies.

In particular, new drug formulations could enable personalized treatment plans, providing more precise therapies tailored to individual patients. Furthermore, combining various treatment methods to enhance therapeutic efficacy, improving the solubility and bioavailability of drugs, and utilizing artificial intelligence to design targeted drugs should all be considered as part of the evolving landscape of cancer therapy using nanocarriers.

## 9 Conclusion

In general, research into novel anti-tumor agents offers new hope for cancer treatment. These innovative formulations have shown significant potential in enhancing drug delivery, reducing side effects and enabling precision therapies. However, challenges such as drug resistance remain prevalent and must be addressed to optimize cancer treatments further. Overcoming these obstacles is crucial for achieving more comprehensive and effective therapies that not only benefit patients but also improve their quality of life and survival rates. Continued research and development are essential to unlocking the full potential of these promising treatments.
